# Physical Deterioration

**Published:** 1906-05-19

**Authors:** 


### PHYSICAL DETERIORATION.

We have lately had occasion to condemn, in no uncertain phraseology, the attempt made by the British Medical Association to extort from candidates for seats in the House of Commons a series of pledges upon debateable points of medical legislation; and we have the more pleasure in recognising, on the part of one of the branches, if not of the parent Association itself, an endeavour to throw its weight, as a representative medical body, upon the side of a systematised effort to promote the better physical culture of the population. The Wandsworth branch of the Association has made arrangements not only to hear an address by Sir Frederic Maurice upon the subject, but also to invite a general audience, both of ladies and gentlemen, to participate in that advantage, and to join, according to their respective degrees of ability, in the practical promotion of the local efforts which will be made in the direction desired. Sir Frederic represents the Association for Physical Culture, which was established some months ago as the direct result of a great meeting held at the Mansion House, and largely due to the efforts of Sir Lauder Brunton—a meeting which was eloquently addressed by the Bishop of Ripon, as well as by Mr. Haldane. As Secretary of State for War, Mr. Haldane may possibly exert very considerable influence in giving effect to the opinions which he announced.

The objects of the Association cover a wide field; but may perhaps be described generally as embracing a desire to combine and organise all existing means of promoting physical culture, and to supply such means in localities where they are insufficient or non-existent. The Association represents a well-founded belief that the cultivation of the physical powers is a branch of education at least as important as the imparting of what is called "knowledge," or the cultivation of the intelligence; together with an approach to some slow and partial recognition of the fact that the *mens sana* is only possible in the *corpus sanum*. Few more hopeful steps have been made in the desired direction than those recently indicated by Mrs. Humphry Ward in her letters to the *Times* on the subject of teaching the children of the poor to play, and of affording them surroundings in which systematised play would be rendered possible; for there can be no question, as a matter of physiological fact, that the ordinary child life of the slums of great cities does not afford a sufficient stimulus to metabolism to provide for the healthy growth and renewal of blood and tissue, or for the healthy accomplishment of secretion. The very attitudes of the slum hobbledehoy or loafer are sufficient to show that his lungs have never been healthfully expanded, and that even such an atmosphere as his surroundings enable him to breathe has never been permitted thoroughly to fill his air-cells.

For the last five-and-thirty years, it is now becoming manifest, the country has been spending enormous sums of money upon a system of education from which the practical benefits to the community are as yet not very manifest. It has given for the outlay a juvenile town population of undersized and badly nourished character, able, indeed, to read election placards, advertisements, and gambling intelligence, but wanting in sound judgment, and possessing but little knowledge of a kind calculated to render them better or more useful citizens. We fear there is only too much resemblance amongst us to the population of Rome during the second century of the Christian era. All students of human development are well aware that the offspring of such a population can only be raised above the level of their parents by conditions which will give them better brains as one portion of better bodies. In the establishment of such conditions the medical profession must be the only capable guides, because they are the only people whose knowledge of physiology enables them to look through details to principles, and thus to avoid a slavish adherence to precedent with regard to objects which are capable of being attained by more than a single method.

We heartily congratulate the Wandsworth members of the Association for their evident willingness to contribute the guidance which is required, and to support by the weight of their authority the measures which it is the business of the Association for Physical Culture to promote. We have compared the state of our urban proletariate to that of the same class of the community in Rome. We wish we could extend the comparison to the more wealthy, and could record modern instances of such magnificent liberality for public purposes as that, for example, of the younger Pliny, a liberality by no means exceptional among his contemporaries, but which has not even been approached at any subsequent period of the world's history.

